# Patient Perspectives with Abbreviated versus Standard Pre-Test HIV Counseling in the Prenatal Setting: A Randomized-Controlled, Non-Inferiority Trial

**DOI:** 10.1371/journal.pone.0005166

**Published:** 2009-04-15

**Authors:** Deborah Cohan, Elvira Gomez, Mara Greenberg, Sierra Washington, Edwin D. Charlebois

**Affiliations:** 1 Department of Obstetrics, Gynecology, & Reproductive Sciences, University of California San Francisco, San Francisco, California, United States of America; 2 Department of Obstetrics and Gynecology, Stanford University, Palo Alto, California, United States of America; 3 Department of Medicine, University of California San Francisco, San Francisco, California, United States of America; Centers for Disease Control and Prevention, United States of America

## Abstract

**Background:**

In the US, an unacceptably high percentage of pregnant women do not undergo prenatal HIV testing. Previous studies have found increased uptake of prenatal HIV testing with abbreviated pre-test counseling, however little is known about patient decision making, testing satisfaction and knowledge in this setting.

**Methodology/Findings:**

A randomized-controlled, non-inferiority trial was conducted from October 2006 through February 2008 at San Francisco General Hospital (SFGH), the public teaching hospital of the City and County of San Francisco. A total of 278 English- and Spanish-speaking pregnant women were randomized to receive either abbreviated or standard nurse-performed HIV test counseling at the initial prenatal visit. Patient decision making experience was compared between abbreviated versus standard HIV counseling strategies among a sample of low-income, urban, ethnically diverse prenatal patients. The primary outcome was the decisional conflict score (DCS) using O'Connor low-literacy scale and secondary outcomes included satisfaction with test decision, basic HIV knowledge and HIV testing uptake. We conducted an intention-to-treat analysis of 278 women – 134 (48.2%) in the abbreviated arm (AA) and 144 (51.8%) in the standard arm (SA). There was no significant difference in the proportion of women with low decisional conflict (71.6% in AA vs. 76.4% in SA, p = .37), and the observed mean difference between the groups of 3.88 (95% CI: −0.65, 8.41) did not exceed the non-inferiority margin. HIV testing uptake was very high (97. 8%) and did not differ significantly between the 2 groups (99.3% in AA vs. 96.5% in SA, p = .12). Likewise, there was no difference in satisfaction with testing decision (97.8% in AA vs. 99.3% in SA, p = .36). However, women in AA had significantly lower mean HIV knowledge scores (78.4%) compared to women in SA (83.7%, p<0.01).

**Conclusions/Significance:**

This study suggests that streamlining the pre-test counseling process, while associated with slightly lower knowledge, does not compromise patient decision making or satisfaction regarding HIV testing.

**Trial Registration:**

ClinicalTrials.gov NCT00503308

## Introduction

Approximately 40% of HIV-infected infants in the United States in 2000 were born to women not diagnosed with their HIV prior to delivery. [1] There are now effective medical therapies to dramatically reduce the risk of perinatal transmission, including anti-retroviral therapy, but this intervention is most effective when women are diagnosed prior to delivery. The Centers for Disease Control and Prevention (CDC), United States Preventive Services Task Force and Institute of Medicine (IOM) have published strong recommendations for universal HIV-antibody testing of pregnant women. [2]–[5] A recent meta-analysis also demonstrated strong evidence supporting routine prenatal HIV testing. [4]

There are currently two common testing strategies. In an opt-out approach, all women are informed of the inclusion of HIV testing in the standard battery of prenatal labs and may decline such testing. On the other hand, standard, opt-in strategies use more traditional voluntary HIV counseling and testing techniques, which typically include pre-test counseling and/or risk assessment. In a recent CDC retrospective analysis, prenatal HIV testing uptake differed widely according to testing strategy used in various regions in the United States and Canada.[3] Prenatal HIV testing was most common in areas with an opt-out approach, with up to 98% of pregnant women testing. In regions using an opt-in approach, testing frequencies were as low as 25%. In September 2006, the CDC published revised recommendations for HIV testing in US healthcare settings. [6] In addition to recommending routine, opt-out prenatal HIV screening, the authors advocated streamlined consenting with removal of requirements for prevention counseling as part of screening and diagnostic HIV testing procedures. While there are population-based studies and other observational studies using historical controls [7] supporting routine, opt-out HIV test counseling, little is known about patient decision making, testing satisfaction and subsequent HIV knowledge with abbreviated counseling.

## Methods

The protocol for this trial and supporting CONSORT checklist are available as supporting information; see Checklist S1 and Protocol S1.

### Ethics Statement

This study was conducted according to the principles expressed in the Declaration of Helsinki. The study was approved by the Institutional Review Board of the University of California San Francisco and San Francisco General Hospital. All patients provided written informed consent for the data collection and subsequent analysis.

This was a randomized-controlled, non-inferiority trial evaluating decisional conflict, HIV knowledge and testing uptake associated with two HIV testing strategies among English and Spanish-speaking pregnant women. The strategies included nurse-performed, standard HIV pre-test counseling (control arm) and abbreviated, pre-test counseling (study arm). The standardized HIV pre-test counseling and consent process took approximately 2–5 minutes and reviewed the definition of HIV, modes of transmission and prevention, interpretation of test results and the benefits of testing. The intervention arm used a 2-sentence script which lasted approximately 30 seconds. See Appendix S1. We hypothesized that decisional conflict and testing uptake would not differ significantly among women in the abbreviated study arm and standard control arm.

Eligible participants included English and Spanish -speaking women aged 16 or older initiating prenatal care at San Francisco General Hospital (SFGH) between October 3, 2006 and September 24, 2007. Women were considered ineligible if they were unable to give informed consent, known to be infected with HIV at initiation of prenatal care, or obtained an HIV test during the index pregnancy prior to initiation of prenatal care at SFGH.

Research staff approached eligible women in the clinic immediately before their initial appointment at the SFGH Women's Health Center. Study staff recruited and obtained written informed consent from all participants and the UCSF Committee on Human Research approved this study. Enrolled participants were randomized to either the standard or abbreviated pre-test counseling strategy. At the onset of the prenatal appointment, the intake nurse gave all pregnant women, regardless of study enrollment, a low-literacy HIV educational brochure to be read at a later time. During the appointment, the nurse administered either the standard or abbreviated HIV pre-test counseling script, documented the woman's decision to take or not take the HIV test and obtained written test consent or refusal from the woman as per California state law at the time of the study. Immediately following the appointment, blinded, bilingual study staff administered a structured questionnaire to each participant. The questionnaire included the 10-item O'Connor Low-Literacy Decisional Conflict Scale (DCS), a 9-item instrument assessing basic HIV knowledge (including HIV as the cause of AIDS, perinatal and non-perinatal modes of transmission, whether people with HIV always look and feel sick and whether there is a cure for HIV), a scale assessing perceived risk of being HIV positive, reasons for testing or not testing and satisfaction with information received and the decision-making process. [8]–[10]

The O'Connor DCS was used in order to assess the level of certainty and satisfaction with the patient's decision to undergo or not undergo HIV testing upon having received the nurse-performed abbreviated or standard pre-test counseling. The scale evaluates the extent to which patients feel informed about a medical decision, whether the patients' assessment of the relative risks and benefits of each decision are consistent with their values, whether patients have enough support and advice to make a decision without pressure from others, and the certainty about the decision. See Table 1. Upon the patients' return for the follow-up clinician visit, typically within 2–4 weeks, blinded study staff administered a second survey following HIV test result disclosure by the medical provider. The second survey included items adapted from Simpson et al. and evaluated overall satisfaction with the consent and results disclosure process as well as the decision to undergo HIV testing. [10] See Appendix S2.

**Table 1:** O'Connor Low-Literacy Decisional Conflict Scale [13] *

Do you know which options are available to you?
Do you know the benefits of each option?
Do you know the risks and side effects of each option?
Are you clear about which benefits matter most to you?
Are you clear about which risks and side effects matter most to you?
Do you have enough support from others to make a choice?
Are you choosing without pressure from others?
Do you have enough advice to make a choice?
Are you clear about the best choice for you?
Do you feel sure about what to choose?

* All questions have 3 response categories: yes, no, unsure

Items are scored as 0 = yes, 2 = unsure, 4 = no.

Scores for each of the 10 items are summed, divided by 2 and multiplied by 25 to calculate the total score.

### Randomization

Study staff generated the randomization scheme using random number tables. HIV consenting scripts, each a single page and identical in weight, were sequentially numbered by study staff and sealed in opaque envelopes which were given to the nurse once the patient consented to participate in the study. Group assignment was not revealed to the study staff until after completion of the study. While participants were informed during the study consent process that they would receive either more or less pre-test counseling, they were not specifically told about their respective group assignment. The nurses were not blinded because they were administering the appropriate script to the patients as per random assignment.

### Statistical methods

We compared differences between women in the control and study arms, including the proportion of women with low decisional conflict (DCS score≤25), mean/median DCS scores, mean/median knowledge scores, the proportion of women reporting overall satisfaction with their decision regarding HIV testing and the percentage of women undergoing HIV testing.

We measured decisional conflict, the primary outcome of the study, using the English or Spanish-language Low-Literacy Decisional Conflict Scale. [8], [9] We calculated a score for all study participants using the Low-Literacy Decisional Conflict Scale which has been validated in many study populations and multiple languages. [11] As has been validated in other studies, we considered a decisional conflict score of 25 or less to be low, corresponding to limited conflict. [11], [12] Other variables measured included demographic characteristics, prior HIV testing history, knowledge about HIV/AIDS, attitudes towards HIV testing, type of provider (physician, midwife or nurse practitioner), perceived risk of being HIV positive and reasons for testing or not testing. We calculated a knowledge score as the percentage of correct answers on the 9-item knowledge scale. We measured the uptake of testing as a proportion of those in each arm who underwent HIV testing. For binary variables, we compared proportions between the 2 groups using the χ^2^ test or Fisher's exact Test. For continuous variables, we assessed for normal distribution using the Kolmogorov-Smirnov test and used the Wilcoxon-Mann Whitney test or Student's T-test as appropriate. A p-value less than 0.05 was considered statistically significant. To assess non-inferiority with respect to the decisional conflict score, we used a one-sided two group t-test of equivalence in means with equal variances with an equivalence limit difference of 5.625. We then evaluated DCS scores and knowledge scores, stratifying by ethnicity, age, primary language spoken and whether the woman had undergone HIV testing in the past. We conducted an intention-to-treat analysis using CONSORT guidelines, including recommendations on reporting of non-inferiority randomized trials. [13], [14]

### Sample Size

Our sample size calculation was based on the primary outcome, decisional conflict score. Because this was a non-inferiority trial, the trial was designed to be able to exclude an actual difference between the abbreviated and standard counseling arms of greater than 5.625 (the non-inferiority margin) with respect to the mean decisional conflict score (DCS) in each group. To calculate our sample size we selected a non-inferiority margin of 5.625 based on investigator judgment of a likely clinically significant difference in DCS. We used a type-I error rate (alpha) of 0.05 with an 80% power, a common standard deviation of 18.75 for the DCS obtained from pilot testing of the DCS instrument, and a one-sided two group t-test of equivalence in means (equal n's) to arrive at 139 patients in each arm for a total of 278 participants. [15], [16]

## Results

**Figure 1:**
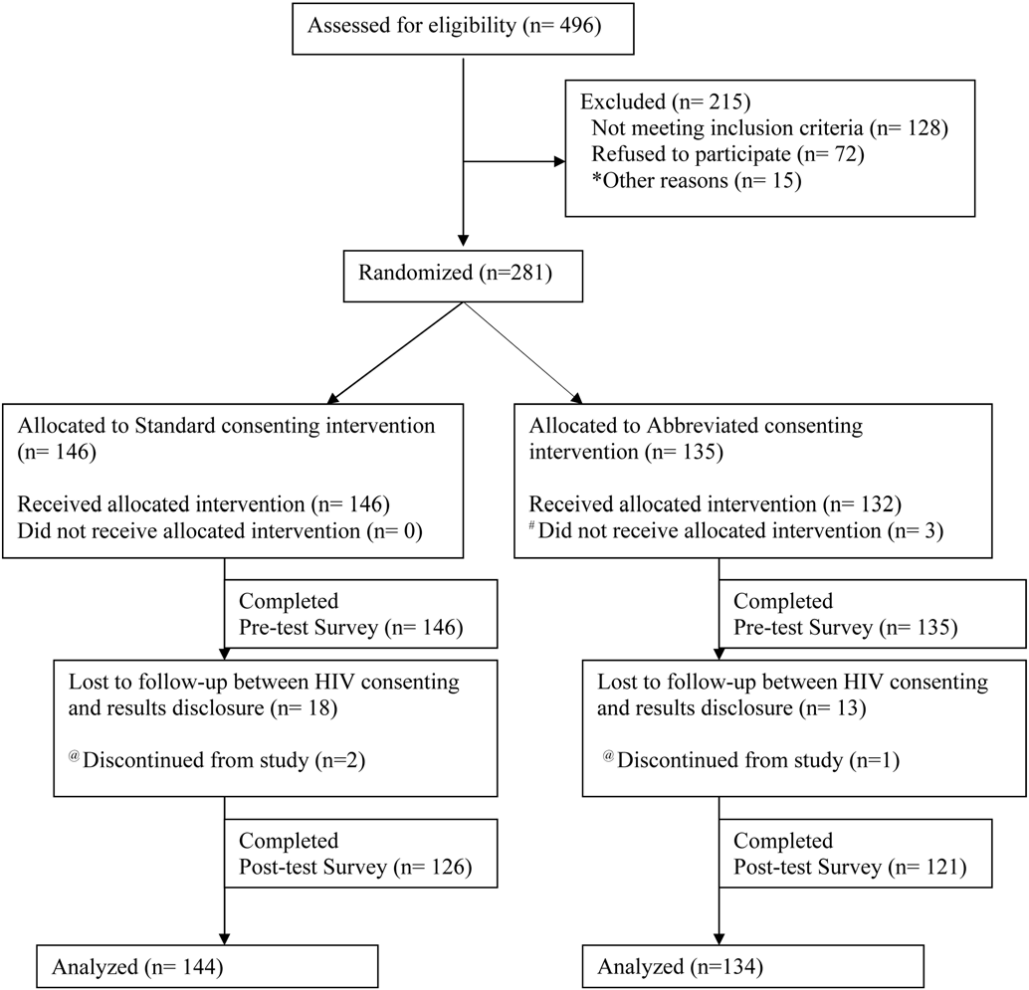
Flow Diagram Legend. * Women who were excluded for “other reasons” included women who initially consented to participate in the study but then changed their mind prior to undergoing the questionnaire. ^#^ Women who received the Standard instead of the Abbreviated consent due to nurse “error”. ^@^ Women were discontinued from study after pre-test survey completed and they were excluded from the analysis. They were identified as being ineligible only after randomization and administration of pre-test survey.

Between October 3, 2006 and September 24, 2007, study staff approached 496 pregnant patients initiating prenatal care at the Women's Health Center at San Francisco General Hospital to participate in the study. We excluded 215 patients from participation in the study. See Figure 1. Of the 281 women randomized to one of the study arms, 3 women were discontinued from the study and excluded from the analysis because they were identified as being ineligible only after randomization. In all 3 cases, the women had been tested for HIV during the index pregnancy prior to the initial prenatal visit at SFGH.

Two hundred seventy eight women were eligible and randomized – 134 (48.2%) in the abbreviated arm (AA) and 144 (51.8%) in the standard arm (SA). Women in the 2 groups were similar at baseline, with no statistically significant differences in age, race/ethnicity, primary language, type of provider, prior HIV testing history, and time since prior HIV test. See Table 2.

**Table 2:** Baseline Characteristics (n = 278)

	Abbreviated	Standard	p-value
	(n = 134)	(n = 144)	
Median age in years (range)	26.0 (17–38)	26.0 (16–42)	.57 *
Ethnicity, No. (%)			
White	8 (6.0)	16 (11.1)	
Black	23 (17.2)	19 (13.2)	
Hispanic/Latina	84 (62.7)	98 (68.1)	.15 #
Asian/Pacific Islander	18 (13.4)	11 (7.6)	
Mixed/Other	1 (0.7)	0 (0)	
Primary Language, No. (%)			
English	62 (46.3)	63 (43.8)	.67
Spanish	72 (53.7)	81 (56.3)	
Provider Type, No. (%)			
Midwife	82 (61.2)	96 (66.7)	
Nurse Practitioner	13 (9.7)	10 (6.9)	.57
Physician	39 (29.1)	38 (26.4)	
Prior HIV testing, No. (%)	80 (59.7)	94 (65.3)	.34
Median # months since prior HIV test (range)	24.0 (0.6–169.1)	17.7 (1.3–176.7)	.17 *
Self-perceived likeliness of HIV infection: Median (range)@	5.0 (1–5)	5.0 (1–5)	.81 *

* Wilcoxon Mann-Whitney test

# Fisher's Exact Test

@ Self-perceived likeliness of HIV infection based on 5 point Liekert scale 1 = very likely→5 = very unlikely [14]

There was no significant difference in the proportion of women with low decisional conflict (71.6% in AA vs. 76.4% in SA, p = .37) See Table 3. Likewise, there were no significant differences in mean DCS scores (mean 19.9 in abbreviated vs. 16.0 in standard arm, p = .17) and the observed mean difference between the groups of 3.88 (95% CI: −0.65, 8.41) did not statistically exceed the non-inferiority margin. There were no differences in DCS scores when stratifying by primary language spoken, ethnicity, age, and prior HIV testing. (Data not shown.) Similarly, women in the 2 arms expressed similar overall satisfaction with their decision to test or not test for HIV (97.8% in AA vs. 99.3% in SA, p = .36). On the other hand, women in AA had significantly lower mean and median knowledge scores (mean = 78.4%) compared to women in SA (mean = 83.7%, p<0.01). In particular, knowledge scores were significantly lower among women in AA who were Spanish-speakers, Latina, ≥ 27 years old or had undergone HIV testing in the past. (Data not shown.) In comparing the 9 knowledge items individually, the only question that women in the standard arm were significantly more likely to answer correctly related to the decreased risk of perinatal HIV transmission through the use of antiretroviral medication during pregnancy. (Data not shown.)

**Table 3:** Decisional conflict, satisfaction, knowledge and testing uptake

	Abbreviated	Standard	p-value
	n = 134 (48.2)	n = 144 (51.8)	
Decisional Conflict Score, No. (%)			
[95% Confidence Interval]			
Low DCS (≤25)	96	110	.37
	(71.6%)	(76.4%)	
	[64.0–79.3]	[69.5–83.3]	
High DCS (>25)	38	34	
	(28.4%)	(23.6%)	
	[20.7–36.0]	[16.7–30.5]	
Mean Decisional Conflict Score (SD)	19.9 (21.0)	16.0 (17.4)	.17
[95% Confidence Interval]	[16.3–23.5]	[13.1–18.9]	
Median Decisional Conflict Score (range)	10.0 (0–90)	10.0 (0–70)	
Mean knowledge score (SD)	78.4 (15.5)	83.7 (13.2 )	<.01
[95% Confidence Interval]	[75.7–81.0]	[81.5–85.9]	
Median knowledge score (range)	77.8 (11.1–100.0)	88.9 (44.4–100.0)	
Testing Uptake, No. (%)	133	139	.12
	(99.3)	(96.5)	
[95% Confidence Interval]	[97.8–100]	[93.5–99.5]	

Overall testing uptake was very high (97.8%) and did not differ significantly between the 2 groups (99.3% in AA vs. 96.5% in SA, p = .12). The most common reason given for testing was “it's a good idea to have it as a routine test” (90.4%). Other reasons given included: “concerned about my own health” (86.0%), “concerned about risks to the baby” (81.2%), “because it was offered” (80.8%) and “clinic staff thought it was a good idea” (67.2%). There were no significant differences in the reasons given by women between the two groups. See Table 4. There were 6 women in our sample who decided not to take the HIV test. The reasons given included: “been in a stable relationship for a long time” (n = 1 AA; n = 4 SA), “partner has been tested elsewhere” (n = 1 AA; n = 3 SA), “tested negative before” (n = 1 AA; n = 3 SA), “not in a high risk group” (n = 2 SA) and “not necessary, as I have no chance of being positive” (n = 2 SA).

**Table 4:** Reasons for testing (n = 272) *

	Abbreviated (n = 133)	Standard (n = 139)	p-value
	No. (%)	No. (%)	
Good idea to have it as a routine test	118 (89.4)	127 (91.4)	.58
Concerned about my own health	114 (86.4)	119 (85.6)	.86
Concerned about risks to the baby	108 (81.8)	112 (80.6)	.79
Because it was offered	106 (80.3)	113 (81.3)	.84
Clinic staff thought it was a good idea	87 (65.9)	95 (68.3)	.67

* Study participants could choose multiple responses.

Of the 278 women enrolled, 247 (89%) returned for a follow-up visit and completed the post-disclosure questionnaire (median time to follow-up visit = 2.7 weeks). Overall, 11.2% of women enrolled in the study were lost to clinical follow-up between initial prenatal visit and medical follow-up visit, 13 women in the abbreviated arm (9.7%) and 18 women in the standard arm (12.5%). At the time of the follow-up survey, all but one participant reported feeling glad about their decision to test or not test (100% in AA vs. 99.2% in SA, p = .33). The only woman reporting not feeling glad about her testing decision was one of the 6 study participants who had not undergone testing. Among the women who responded they were glad about their decision regarding testing, 2 (0.8%) were women who had decided not to take the test. There were no significant differences among women in the 2 arms reporting their comfort waiting for test results (91.7% in AA vs. 91.1% in SA, p = .85) as well as stating they knew as much about HIV/AIDS as they wanted to (74.4% in AA vs. 77.8% in SA, p = .53) and had their questions related to HIV answered (97.5% in AA vs. 95.1% in SA, p = .32).

## Discussion

This study suggests that streamlining the pre-test consenting process, while associated with lower knowledge, does not compromise patient decision making or satisfaction regarding HIV testing. Studies in Scotland have shown that provider-performed HIV counseling and testing is associated with increased uptake of HIV testing in the perinatal setting and is equally acceptable as testing strategies involving passive HIV testing education. [17] Moreover, a cross-sectional study of English and Spanish-speaking prenatal patients found that nearly 70% of women supported “routine” HIV testing (“all pregnant women should be given this test”) as compared to 27% who supported “elective” testing (“only on women who want it and who give their permission to have the test”). [18] This support for routine prenatal HIV testing was comparable to these women's attitudes towards routine rubella screening (63%) and routine gonorrhea/chlamydia screening (77%). While the O'Connor Decisional Conflict Scale has been used in studies evaluating a vast range of health decisions from breast cancer treatment and autologous pre-donation in cardiac surgery to PSA screening, [9], [19]–[25] [9], [19]–[25] we know of only one other study utilizing this scale to assess patient perspectives on HIV testing. [26] In this study of 46 pregnant women undergoing rapid HIV testing on Labor and Delivery at our institution, we found that 89% of the women reported feeling satisfied with their testing experience, and 82.6% of women reported no decisional conflict (score≤25) in making decisions for rapid testing, similar to what was seen in this current study.

While knowledge scores were statistically significantly lower among women randomized to the abbreviated study arm, knowledge scores were overall high among women in both arms of the trial. The overall high scores were notable and reassuring given the ethnic diversity and low literacy of our clinic population. [27], [28] The difference in knowledge scores was driven primarily by women in the abbreviated arm being less likely to know that HIV-infected pregnant women can take medication to decrease the risk of perinatal HIV transmission. As more institutions and prenatal practices institute the CDC recommended abbreviated HIV pre-test counseling, our data identify gaps in knowledge among low-income, ethnically-diverse pregnant patients and may assist in guiding population-level campaigns that will likely replace the one-on-one pre-test counseling session as a source of patient education.

While we attempted to minimize bias with the use of blinded study staff, standardized protocols and validated instruments, our study had limitations. As with most surveys assessing patient satisfaction and perceptions, participants may have been biased towards reporting positive attitudes towards testing and limited decisional conflict, despite reassurances about confidentiality of responses and the use of blinded study staff. The slight, though statistically insignificant, higher mean decisional conflict score seen among women in the abbreviated arm suggest that responses may indeed have been truthful. While we did not monitor in real time the nurses administering the standard and abbreviated counseling scripts, we utilized standardized protocols and scripts and trained the nurses during study implementation and conducted on-going education to the nurses about study protocols. Nonetheless, there may have been deviations from the assigned consenting script that went unnoticed. Moreover, efforts to optimize the uptake of prenatal HIV testing in this clinical setting were instituted prior to initiation of this study even with the use of more traditional standard pre-test counseling by nurses as we have reported elsewhere. [29] Given the very high acceptance of prenatal HIV testing under non-study conditions at our clinic, our results may not be generalizable to other clinical settings with lower baseline uptake of HIV testing. Similarly, our results may not be generalizable to areas with less developed HIV educational and public health campaigns as compared to San Francisco. Lastly, participants were, by definition, willing to give additional time and effort to participate in this research. As such, participants may have differed in significant and clinically relevant ways from non-participants. In particular, participants may have been more agreeable to health interventions as compared to non-participants and, thus, may have biased the data toward increased satisfaction and minimized the differences between the 2 arms of the study. This bias may also limit the generalizability of our results to populations of pregnant women who would not otherwise participate in research. While selection bias may limit the generalizability of our results, study participation was very high – only 14.5% of women approached for enrollment refused to participate.

Despite these potential limitations, this study adds to the growing body of literature evaluating best practices in integrating routine HIV testing into medical settings. While there were no differences seen in testing uptake in our study, this streamlined approach to pretest consenting would likely facilitate the systematic implementation the CDC guidelines recommending universal HIV testing of pregnant women. As such, abbreviated HIV testing strategies may be associated with increased HIV testing uptake on a population level without jeopardizing patients' decision-making process. There are numerous states in the U.S. that still require detailed pre-test counseling and consenting prior to prenatal HIV testing. [30] These data support the elimination of such requirements and should bolster legislative efforts to update HIV testing laws aimed at decreasing the burden on health-care providers and reducing overall barriers to HIV testing.

## Supporting Information

Appendix S1 (0.04 MB DOC)

Appendix S2 (0.04 MB DOC)

Checklist S1 CONSORT Checklist
(0.07 MB DOC)

Protocol S1 Trial Protocol
(0.05 MB DOC)
