# Supplementary material for: Patient Perspectives with Abbreviated versus Standard Pre-Test HIV Counseling in the Prenatal Setting: A Randomized-Controlled, Non-Inferiority Trial
Source: PLoS One. 2009 Apr 15;4(4):e5166. doi: 10.1371/journal.pone.0005166 (PMC2666158; doi:10.1371/journal.pone.0005166)
Supplement: Appendix S1 — (0.04 MB DOC) [file pone.0005166.s001.doc]

Appendix S1.

**Abbreviated Pre-test Counseling Script**

We do the following tests on all pregnant women as part of routine prenatal care.

- Blood type and blood count
- Gonorrhea, chlamydia, syphilis, hepatitis B, tuberculosis and HIV (Human Immunodeficiency Virus)
- Verification of vaccination against Rubella (German measles)
- Blood sugar
- Urine testing for infection
- Pap smear

We will perform an HIV test on you during pregnancy unless you refuse the test.

Do you have any questions?

**Standard Pre-test Counseling Script**

We recommend that all pregnant women get tested for HIV (Human Immunodeficiency Virus) during pregnancy. We do an HIV test as part of routine prenatal care. We will perform an HIV test on you during pregnancy unless you refuse the test.

HIV is the virus that causes AIDS.

People with HIV can pass it to others even if they look and feel healthy.

A person with HIV can pass the virus through sex without a condom or sharing needles. A mother with HIV can pass the virus to her baby during pregnancy, childbirth, or breastfeeding.

A mother with HIV can take medicine during pregnancy and childbirth to lower the chance of passing the virus to her baby. This medicine can also help HIV-infected women stay healthy and live longer.

We check for HIV with a blood test. It can take up to 3 months after getting the virus for someone’s blood to show HIV.

*****A *NEGATIVE* test means that either

You do not have HIV, or

You may have gotten HIV recently and the virus has not shown up yet on the test.

*****A *POSITIVE* test means that you have HIV, and your medical provider will offer you treatment options.

HIV testing is voluntary and confidential. You will still get regular prenatal care even without the test but we strongly recommend that you get tested. No one but the providers caring for you will see your results.

We report all positive HIV test results to the Department of Public Health. The results are always kept private and confidential.

Do you have any questions?
