# Supplementary material for: Patient Perspectives with Abbreviated versus Standard Pre-Test HIV Counseling in the Prenatal Setting: A Randomized-Controlled, Non-Inferiority Trial
Source: PLoS One. 2009 Apr 15;4(4):e5166. doi: 10.1371/journal.pone.0005166 (PMC2666158; doi:10.1371/journal.pone.0005166)
Supplement: Appendix S2 — (0.04 MB DOC) [file pone.0005166.s002.doc]

Appendix S2. Study Flow

| Screening, consent randomization | 🡲 | Nurse intake with abbreviated or standard HIV pre-test counseling. Patient given HIV educational brochure. | 🡲 | Blinded study staff administer low-literacy decisional conflict scale and knowledge survey | 🡲 | Appointment with midwife, physician or nurse practitioner (results disclosure, 2-4 weeks later) | 🡲 | Blinded study staff administer follow-up survey |
| --- | --- | --- | --- | --- | --- | --- | --- | --- |
