# Supplementary material for: Patient Perspectives with Abbreviated versus Standard Pre-Test HIV Counseling in the Prenatal Setting: A Randomized-Controlled, Non-Inferiority Trial
Source: PLoS One. 2009 Apr 15;4(4):e5166. doi: 10.1371/journal.pone.0005166 (PMC2666158; doi:10.1371/journal.pone.0005166)
Supplement: Protocol S1 — Trial Protocol (0.05 MB DOC) [file pone.0005166.s004.doc]

**Trial Protocol**

**Synopsis**

We propose a randomized controlled, non-inferiority trial to evaluate the effectiveness of two HIV testing strategies among 278 pregnant women seeking prenatal care at San Francisco General Hospital. The two strategies will include pretest/post-test counseling using prenatal care nurses and medical providers (control arm; current standard of care at San Francisco General Hospital [SFGH]) and an abbreviated pretest/post-test CDC recommended strategy using prenatal care nurses and medical providers (study arm). We hypothesize that the abbreviated CDC recommended strategy will be equally acceptable to patients as the standard strategy currently used. Eligible participants will include Spanish or English-speaking women seeking prenatal care at San Francisco General Hospital over the course of approximately one year. Eligible women presenting to SFGH physician or midwife prenatal clinics will be recruited, consented and randomized by study staff to either the standard or abbreviated testing strategy. The outcomes studied will be patient satisfaction and the proportion of study participants who undergo HIV testing. Satisfaction will be measured using the validated Decisional Conflict scale.

**Specific Aims**

1. To compare the acceptability of standard and abbreviated HIV counseling/testing strategies among a sample of low- income, urban, ethnically diverse prenatal patients.
2. To create an abbreviated HIV counseling/testing protocol compatible with the new California prenatal HIV testing law (AB 1676) that can be used in prenatal settings throughout the state.
3. To compare the proportion of enrolled pregnant women tested for HIV by the standard, risk-based vs. abbreviated counseling/testing strategy.
4. To compare the proportion of HIV-tested pregnant women receiving their HIV test results by the standard vs. abbreviated counseling/testing strategy.

**Background and Significance**

Approximately 40% of HIV-infected infants in the United States in 2000 were born to women not diagnosed with their HIV prior to delivery. (1) There are now effective medical therapies to prevent perinatal transmission, including anti-retroviral therapy, but this requires diagnoses of maternal HIV prior to delivery. Both the Centers for Disease Control and Prevention (CDC) and Institute of Medicine (IOM) have published strong recommendations for universal HIV-antibody testing of pregnant women. (8-12) There are currently two common testing strategies; in an opt-out approach, women are informed of the inclusion of HIV testing in the standard battery of prenatal labs and may decline such testing, whereas opt-in strategies use more traditional voluntary HIV counseling and testing techniques, with a specific informed-consent process. In a recent CDC retrospective analysis, prenatal HIV testing frequencies differed widely according to testing strategy throughout the United States and Canada. (16) Prenatal HIV testing was most common in areas with an opt-out approach, up to 98%, whereas regions using an opt-in approach had testing frequencies as low as 25%. While there are population-based studies such as this one and other observational studies using historical controls (18) supporting opt-out testing, there have been no randomized trials directly comparing opt-in and opt-out strategies. In addition, studies have shown that provider-performed HIV counseling and testing increases uptake of HIV testing in the perinatal setting, and is equally acceptable as testing strategies involving passive HIV testing education. (20)

The California legislature and former Governor Gray Davis passed Assembly Bill (AB) 1676, which requires all prenatal providers to inform their patients of their intent to include HIV testing as part of the routine battery of prenatal labs (similar to opt-out) but also requires pre-test counseling and written informed consent (similar to opt-in). Thus while opt-out HIV testing is restricted in California, an abbreviated provider-performed HIV counseling and testing strategy would be possible.

**Preliminary Studies**

A retrospective chart review of pregnant women accessing prenatal care at SFGH Women’s Health Clinic (WHC) showed that due to the change in standard of care at SFGH, from a dedicated HIV counseling and testing service to provider-performed counseling and testing, prenatal HIV testing uptake at SFGH has increased from 52% in 2003 to 93% as of April 2006. In a cross-sectional study of prenatal patients at SFGH WHC, nearly 70% of women surveyed supported routine testing as compared to 27% who supported elective testing. This support for routine prenatal HIV testing was comparable to these women’s attitudes towards routine rubella screening (63% support) and routine gonorrhea/chlamydia screening (77% support). The study we propose would be the natural next step in more specifically evaluating the routinization and streamlining of HIV testing in the prenatal care setting through the use of a rigorous randomized methodology.

**Study Design**

This will be a randomized controlled, non-inferiority trial comparing two HIV testing strategies among English and Spanish-speaking patients presenting for prenatal care at SFGH WHC over the course of approximately one year. Eligible participants will be randomized by study personnel to either standard HIV counseling and testing (control arm) or abbreviated counseling and testing (study arm). See Appendix 1. Participants will receive a standard prenatal HIV testing brochure, undergo one of two HIV counseling/testing strategies, submit blood for an HIV-1 antibody test, and will follow-up with their medical provider for HIV test results. Upon completion of counseling, participants will undergo a short, structured (Pre-test) questionnaire administered by study staff. Following testing and receipt of results, participants will complete a second (Post-test) structured questionnaire administered by study staff. Eligible women presenting to SFGH physician or midwife prenatal clinics will be recruited, consented and randomized by study staff to either the standard or abbreviated testing strategy. The outcomes studied will be patient satisfaction and the proportion of study participants who undergo HIV testing. Potential confounder variables to be measured will include demographic characteristics, prior HIV testing history, knowledge about HIV/AIDS, attitudes towards HIV testing, HIV test result, and type of provider (physician versus midwife).

All procedures will take place in SFGH Women’s Health Clinic. Time required of the subjects outside of routine prenatal care visits includes approximately 10 minutes for recruitment and randomization, 5-10 minutes for the pre-test questionnaire, and 5 minutes for the post-test questionnaire. If the patient is lost to follow-up and can no longer be reached upon visiting the clinic, the follow-up interview will be attempted to be conducted via telephone. We will specifically ask the participant permission on whether or not we can leave a simple message on her voicemail if she is lost to follow-up.

**Recruitment**

All English and Spanish-speaking pregnant patients initiating prenatal care at SFGH Women’s Health Center are eligible to participate. We will offer study enrollment to English and Spanish speaking pregnant patients initiating prenatal care at SFGH WHC. We will include women who are 16 years or older and are capable of providing informed consent. We will exclude women who do not speak either English or Spanish, women who are not able to give informed consent, women younger than 16 years old and women who obtained an HIV test during the index pregnancy prior to initiation of prenatal care at SFGH. In addition, we will exclude pregnant women who are known to be infected with HIV at initiation of prenatal care at SFGH.

We will be posting flyers in the Women’s Health Clinic so that patients will be aware of the study. We will also be providing only a small monetary incentive for their participation. We will reimburse the subjects $10 for their participation in the study if such funding is available at the time of subject recruitment. Specifically, we will reimburse $5 at the completion of the pre-test questionnaire and $5 at the completion of the post-test questionnaire. Additionally, bilingual/bicultural research staff will be conducting recruitment, administering the study consents and surveys. We will also be using a validated low-literacy scale to measure satisfaction.

**Informed Consent**

We will obtain written informed consent from each study participant. The consent will review the purpose of the research, what each participant will undergo, the time that will be required, the follow-up provided for positive tests, the risks and benefits, the protocols for maintaining confidentiality, and the voluntary nature of participation. This will take place at the nursing intake visit as mentioned above, by the research staff member recruiting the subject’s participation. The location is at the Women’s Health Center (5M) at SFGH. Subjects will be given time to consider study participation and research staff will be readily available to answer questions from potential subjects. In addition, all eligible patients will receive the standard prenatal HIV testing brochure. All women electing to undergo HIV testing will also sign the HIV testing consent form consistent with California state law.

**Statistical Analysis**

We will measure the receipt of results as a proportion of those undergoing testing who receive their results before the onset of labor. We will compare the proportions between the 2 groups using the χ² test. We will calculate a satisfaction score for all study participants using the Decisional Conflict Scale which has been validated in many study populations. (20) We will compare mean satisfaction scores between the 2 groups using the Student t-test (if the distribution is parametric) or Wilcox rank-sum test (if the distribution is non-parametric). Lastly, we will conduct multivariate logistic regression to assess independent predictors using STATA SE8. We will test for interaction and stratify the multivariate analysis accordingly. We will conduct a strict intention-to-treat analysis. We will develop the data collection forms with the date and alphanumeric code on each page. Each variable will be named and coded. We will utilize a Microsoft ACCESS relational database. We will pretest the data management system with “dummy” data. We will promptly enter the data after collection and set criteria within the ACCESS database to minimize the likelihood of incorrect data entry. Once all data is entered, we will edit the database, checking for missing data, illogical data and outliers. We will log any changes made in the database during the editing process and freeze the final database before importing the data into statistical analysis software. We will then analyze and archive the database.

**Sample Size**

Our sample size calculation was based on the primary outcome, decisional conflict score. Because this was a non-inferiority trial, the trial was designed to be able to exclude an actual difference between the abbreviated and standard counseling arms of greater than 5.625 (the non-inferiority margin) with respect to the mean decisional conflict score (DCS) in each group. To calculate our sample size, we used a type-I error rate (alpha) of 0.05 with an 80% power, a common standard deviation of 18.75 for the DCS, and a one-sided two group t-test of equivalence in means (equal n's) to arrive at 139 patients in each arm for a total of 278 participants. ^20, 21^ Assuming 75% participation as was seen in a 1997 study of the same patient population (i.e. those accepting enrollment and randomization), we will need to approach 371 women overall.

Appendix 1.

**Abbreviated Pre-test Counseling Script**

We do the following tests on all pregnant women as part of routine prenatal care.

- Blood type and blood count
- Gonorrhea, chlamydia, syphilis, hepatitis B, tuberculosis and HIV (Human Immunodeficiency Virus)
- Verification of vaccination against Rubella (German measles)
- Blood sugar
- Urine testing for infection
- Pap smear

We will perform an HIV test on you during pregnancy unless you refuse the test.

Do you have any questions?

**Standard Pre-test Counseling Script**

We recommend that all pregnant women get tested for HIV (Human Immunodeficiency Virus) during pregnancy. We do an HIV test as part of routine prenatal care. We will perform an HIV test on you during pregnancy unless you refuse the test.

HIV is the virus that causes AIDS.

People with HIV can pass it to others even if they look and feel healthy.

A person with HIV can pass the virus through sex without a condom or sharing needles. A mother with HIV can pass the virus to her baby during pregnancy, childbirth, or breastfeeding.

A mother with HIV can take medicine during pregnancy and childbirth to lower the chance of passing the virus to her baby. This medicine can also help HIV-infected women stay healthy and live longer.

We check for HIV with a blood test. It can take up to 3 months after getting the virus for someone’s blood to show HIV.

*****A *NEGATIVE* test means that either

You do not have HIV, or

You may have gotten HIV recently and the virus has not shown up yet on the test.

*****A *POSITIVE* test means that you have HIV, and your medical provider will offer you treatment options.

HIV testing is voluntary and confidential. You will still get regular prenatal care even without the test but we strongly recommend that you get tested. No one but the providers caring for you will see your results.

We report all positive HIV test results to the Department of Public Health. The results are always kept private and confidential.

Do you have any questions?
